# Proteomic identification of proteins differentially expressed following overexpression of hTERT (human telomerase reverse transcriptase) in cancer cells

**DOI:** 10.1371/journal.pone.0181027

**Published:** 2017-07-13

**Authors:** Rishi Kumar Jaiswal, Pramod Kumar, Amod Sharma, Deepak Kumar Mishra, Pramod Kumar Yadava

**Affiliations:** Applied Molecular Biology Laboratory, School of Life Sciences, Jawaharlal Nehru University, New Delhi, India; University of Newcastle, UNITED KINGDOM

## Abstract

Reverse transcriptase activity of telomerase adds telomeric repeat sequences at extreme ends of the newly replicated chromosome in actively dividing cells. Telomerase expression is not detected in terminally differentiated cells but is noticeable in 90% of the cancer cells. hTERT (human telomerase reverse transcriptase) expression seems to promote invasiveness of cancer cells. We here present proteomic profiles of cells overexpressing or knocked down for hTERT. This study also attempts to find out the potential interacting partners of hTERT in cancer cell lines. Two-dimensional gel electrophoresis (2-DE) of two different cell lines U2OS (a naturally hTERT negative cell line) and HeLa revealed differential expression of proteins in hTERT over-expressing cells. In U2OS cell line 28 spots were picked among which 23 spots represented upregulated and 5 represented down regulated proteins. In HeLa cells 21 were upregulated and 2 were down regulated out of 23 selected spots under otherwise identical experimental conditions. Some heat shock proteins viz. Hsp60 and Hsp70 and GAPDH, which is a housekeeping gene, were found similarly upregulated in both the cell lines. The upregulation of these proteins were further confirmed at RNA and protein level by real-time PCR and western blotting respectively.

## Introduction

Cancer cells have unlimited proliferation potential. One way of acquiring this involves reactivation of a specialized reverse transcriptase called telomerase which solves the end replication problem by adding telomeric repeats on to the 3’ ends of template strands so as to minimize on attrition of the lagging strands at their terminal 5’ ends. Telomerase activity is found to be high in nearly 90% of cancerous cells as compared to normal differentiated somatic cells which do not have detectable telomerase activity. The telomerase basically consists of six main subunits viz. hTERT (human telomerase reverse transcriptase), dyskerin, p23, Hsp90, hTERC (human telomerase RNA component) and TEP1 (telomerase-associated protein 1) [1]. Out of these six subunits, hTERT and hTERC can reconstitute the classical telomere lengthening in vitro and also perform many extracurricular functions of regulatory nature in vivo [2]. Stabilization of telomere length of fibroblast and other cell types is achieved by ectopic expression of hTERT in these cell lines which thus acquire infinite replicative potential [3]. Immortalization of both cancer cells and normal stem cells can be achieved by overexpression of telomerase [4–6]. Moreover, knowing the main roles of telomerase in cancer cells would be helpful in the development of exact therapeutic strategies on the basis of telomerase inhibition [7,8]. Here, we have studied proteomic profile of cells following hTERT overexpression in two different cell lines viz., the human osteosarcoma cell line U2OS, which is telomerase negative and HeLa, a cervical cancer cell line that has its own telomerase activity.

## Materials and methods

### Cell culture

Two cell lines Viz., 1) U2OS (an hTERT negative human osteosarcoma cell line) and 2) HeLa cells (an hTERT expressing cervical cancer cell line) were obtained from National Centre for Cell Science, Pune and grown in Dulbecco’s modified Eagle’s medium (DMEM; Hyclone, South Logan Utah,) with 10% fetal bovine serum (FBS) (Himedia). Cells were maintained at 37°C and 5% CO2 in a humidified CO2 incubator.

### Transfection of cells and establishment of stable cell lines overexpressing hTERT

One day before transfection HeLa and U2OS cells were seeded in 6 well plates and grown in Dulbecco’s modified Eagle’s medium (DMEM) containing 10% FBS and antibiotics (penicillin/ streptomycin). Two micrograms each of pBABE-puro empty vector and pBABE-puro hTERT obtained from Addgene and 2 μg of each were transfected in to HeLa cells and U2OS cells by using lipofectamine 3000 (Invitrogen). After 48hrs, transfected cells were selected by using 2μg/ml of puromycin and finally maintained in 1μg/ml of puromycin. Total protein was extracted at passage number 5 for two-dimensional gel electrophoresis.

### Wound healing assay

Cell migration required for healing artificially created wound was assayed at 0, 12, 24, 36 and 48 hrs for hTERT overexpressing HeLa and U2OS cells. Briefly both HeLa and U2OS cells were separately seeded in 2 wells of a 6 well plate and cultured until confluency. Then by using a pipette tip we made a straight scratch, simulating a wound. The plates were washed gently and fresh DMEM replaced with supplements (serum, antibiotics). The cells were observed by phase contrast microscopy.

### Two dimensional gel electrophpresis

We profiled cellular proteins after the overexpression of hTERT in HeLa and U2OS cells. 2D-Gel electrophoresis was performed as reported by Diao S et al [9]. Briefly, isoelectric focusing (IEF) was performed using an Ettan IPGphore 3 apparatus (GE healthcare) and using the nonlinear IPG strips of 13 cm in the pH range of 3.0–10.0. A total of 250 μg protein was diluted to 250 μl in a rehydration buffer (7M urea, 2M thiourea, 4% CHAPS, 1% DTT, 0.5% IPG buffer and some traces of bromophenol blue) and the rehydration step was continued for 16 h at room temperature. IEF was run following a step-wise voltage increase procedure in the following order, 500 V for 5 hrs, 1000 V for 1 h and 8000 V for 3.5 h. After IEF, the IPG gel strips were placed in an equilibration buffer (5M urea, 10% SDS, 10% glycerol, 1.5 M Tris-HCL, pH 8.8 and some traces of bromophenol blue) 1 and 2 for 15 min each and then kept in SDS-page running buffer for 5 min. Equilibration buffer 1 contained 1% DTT while equilibration buffer 2 contained 2.5% iodoacetamide. Separation in the second dimension was performed by SDS-polyacrylamide (12%) gel elctrophoresis at constant voltage of 120 volt till the bromophenol blue dye front reached the lower end of the gels. Gels were fixed for 1 h in fixing solution (50% methanol and 10% acetic acid) and stained in colloidal Coomassie G-250 stain (that is compatible with downstream MS analysis, as peviously described [10] for 3 h, and then destained with 10% acetic acid. The images were scanned with a scanner (Ettan IPGphor3). Images were analysed by using ImageMaster 2D Platinum v7.0 gel analysis software-(GE Healthcare Life Sciences). After analysis of spots and normalizing for the background we excised 28 spots of our interest from the U2OS and 23 from HeLa and submitted them for mass analysis.

### In-gel protein digestion and MALDI-TOF-TOF/MS analysis

Trypsin digestion of the excised bands were done according to Shevchenko et al. [11]. Briefly, after distaining, the gel was washed twice with mili-Q water and the spots of interest excised from the gel and cut into 1mm cubes. These small 1mm cubes of gels were transferred to a 1.5 ml microcentrifuge tube pre-rinsed with 100% acetonitrile. Gel particles were further washed with a solution of 100 mM ammonium bicarbonate in 100% acetonitrile and water. After 15 minute of incubation on a rotatory shaker supernatant was discarded and this step was repeated till completion of destaining. After destaining all the remaining liquids were removed and enough acetonitrile was added to cover the gel particles which let the particles to shrink together. Acetonitrile was removed completely and gel particles were dried down in a vacuum centrifuge at room temperature. Further gel particles were swelled in a solution of 50 μl each of 10 mM DTT and 100 mM ammonium bicarbonate and incubated for 45 minute at 56°C. After this incubation, tubes were cooled at room temperature and excess liquid removed and replaced quickly by same volume as above of freshly prepared solution of 55 mM iodoacetamide in 100 mM ammonium bicarbonate and further incubated for 30 min at room temperature. The gel was washed again with a solution of acetonitrile and ammonium bicarbonate and dried down in a vacuum centrifuge. Enough sequencing grade modified trypsin was added in the tube and incubated at 37°C for 30 minutes. 5μl of 25 mM ammonium bicarbonate was added to keep the gel moistened. Trypsin added tubes were further incubated at 37°C overnight. Next day supernatants were collected in new microfuge tubes and 10 μl of 1% TFA and 10 μl of 100% acetonitrile were added to the gel and the mix sonicated for 20 min at room temperature. Supernatants were taken and pooled and further dried in Speed vac and submitted for mass analysis.

### RNA isolation and quantitative real-time PCR (qRT-PCR)

Total RNA was isolated from U2OS and HeLa cells using TRIzol reagent (Sigma). cDNA was synthesized by using reverse transcription kit (Thermofisher) according to the manufacturer’s protocol. 1 μg cDNA was used as template for PCR reaction using gene specific primers. The real-time primers sequences are given in Table 1. Real Time PCR conditions were: 15 sec at 95°C for denaturation, 1 min at 60°C for both annealing and elongation over 40 cycles in Applied Biosystems 7500. Data were normalized with reference to actin used as endogenous control.

**Table 1:** Primers used for PCR-based assays.

Gene name	Real-time primers sequences (5’-3’)
HSP60	Forward primer- TGCCAATGCTCACCGTAAG
	Reverse primer- ACTGCCACAACCTGAAGAC
HSP70	Forward primer- ACCAAGCAGACGCAGATCTTC
	Reverse primer- CGCCCTCGTACACCTGGAT
HSP90	Forward primer- ACTACACATCTGCCTCTGGTGATGA
	Reverse primers- TGTTTCCGAAGACGTTCCACAA
hTERT	Forward primer- CGGCGACATGGAGAACAAG
	Reverse primers- CCAACAAGAAATCATCCACCAAA
GAPDH	Forward primer- GTCTTCACCACCATGGAGAAGGCT
	Reverse primers- CATGCCAGTGAGCTTCCCGTTCA
ACTIN	Forward primer- GGCACCCAGCACAATGAAG
	Reverse primers- GCCGATCCACACGGAGTACT

### Western blotting

Western blotting was performed as previously described [12]. Stable U2OS and HeLa cells carrying pBABE-puro empty vector and pBABE-hTERT were lysed in RIPA buffer, (GCC biotech). Cell lysates were quantified by Bradford assay and 40 μg of total protein was separated by SDS polyacrylamide gel electrophoresis. Proteins resolved on SDS-PAGE gels were further transferred by making sandwitch of (-ve pole) transfer pads-two Whatman filter paper-gel-Polyvinylidene difluoride (PVDF) membrane (Millipore)-two Whatman filter paper-transfer pads (+ve pole) clamped between transfer sheets. The whole assembly was fitted in the transfer apparatus in a way that the membrane should be on the positive pole while the gel should be on negative pole which facilitates the migration of negatively charged proteins towards positive pole. Whole assembly is run at 80 volts of constant voltage at 4 degree for 2 hrs. The blots were kept in blocking buffer (5% skimmed milk in 1xPBST) for two hours on a reciprocating shaker. After blocking, blots were incubated with primary antibody against hTERT (Santa Cruz, USA, Sc-393013, lot# F0716), β-actin (Santa Cruz, USA, Sc-47778, Lot# 12208), HSP90 (Enzo-Life sciences, ADI-SPA-844-F), HSp70 (Enzo-Life sciences, ADI-SPA-757-F), GAPDH and HSP 60 (Enzo-Life sciences, ADI-SPA-806-F), followed by incubation with secondary antibodies i.e., Goat anti-rabbit immunoglobulin G (IgG), horseradish peroxidase (HRP)-linked antibody (dilution, 1:5,000, Bangalore Genei, 114038001A) and Goat anti-mouse immunoglobulin G (IgG), horseradish peroxidase (HRP)-linked antibody (dilution, 1:5,000, Bangalore Genei, 114068001A). Luminata^™^ Forte western HRP substrate was used for band visualization according to the manufacturer's protocol. β-actin was used as an internal control for protein expression. Quantification of protein expression was done by ImageJ software. (Primary data related to methodology and results presented in this paper may be viewed in S1 File)

## Results

### Overexpression of hTERT in U2OS and HeLa cell line

**Fig 1:**
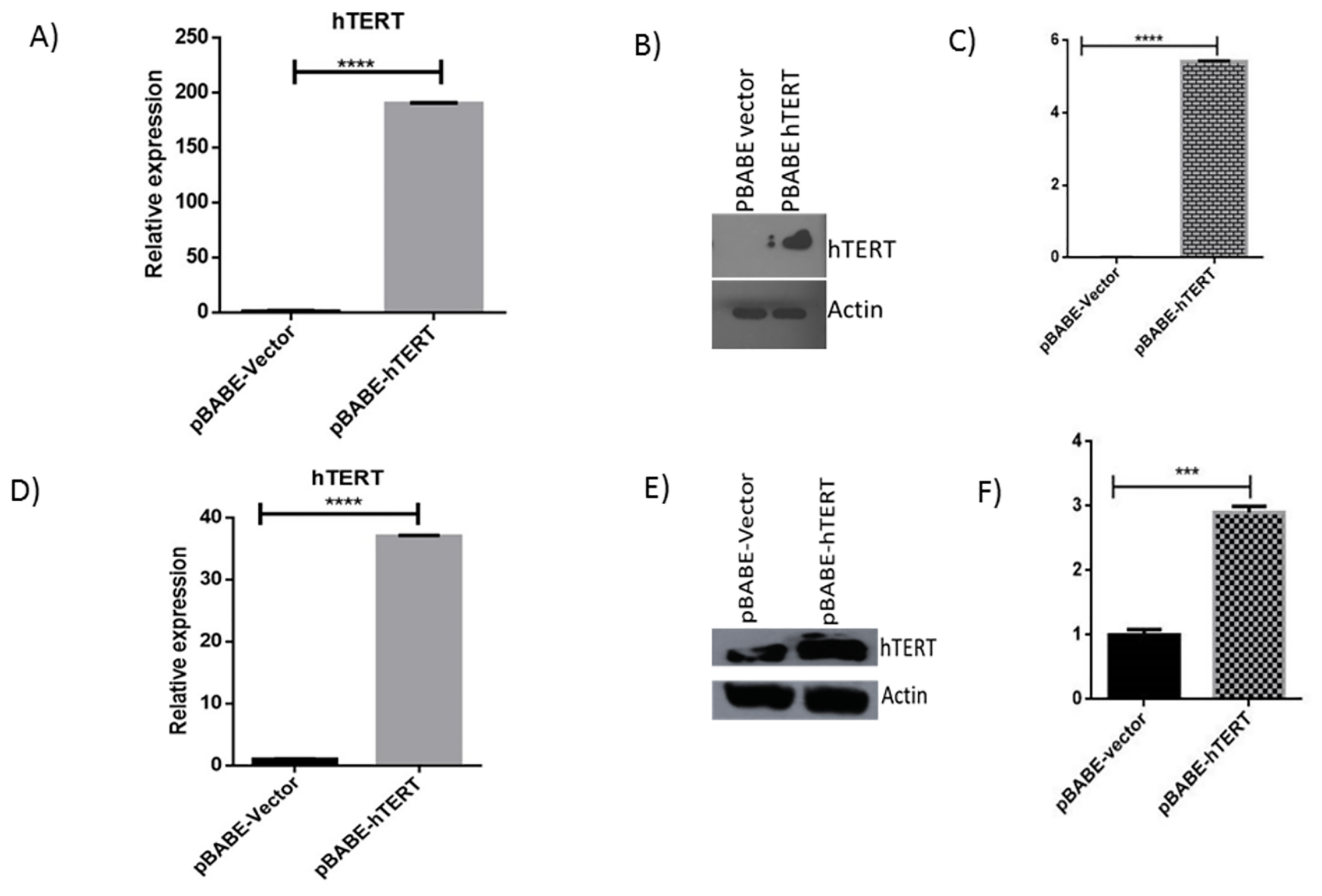
hTERT overexpression in HeLa and U2OS cell lines. hTERT is overexpressed in U2OS and HeLa cell lines. (A & D) mRNA level of hTERT in U2OS & HeLa cell lines was determined by quantitative real-time PCR. (B & E) Western blotting confirms overexpression of hTERT in U2OS and HeLa cell line. (C & F) Histograms depict densitometric quantification of the hTERT overexpression of three corresponding independent Western blot experiments in U2OS and HeLa cell lines.

Overexpression of hTERT in HeLa and U2OS cells was confirmed by qRT-PCR and western blotting. Remarkably, hTERT mRNA expression was upregulated to approximately 180 fold in U2OS cells transfected with pBABE-puro-hTERT in comparison to vector transfected HeLa cells (Fig 1A) while in HeLa it is upregulated to 36 fold (Fig 1C). Overexpression of hTERT in U2OS and HeLa cell lines was futher confirmed by western blotting (Fig 1B and 1D).

### Overexpression of hTERT in U2OS and HeLa cell line enhances the migration rate of these cells

**Fig 2:**
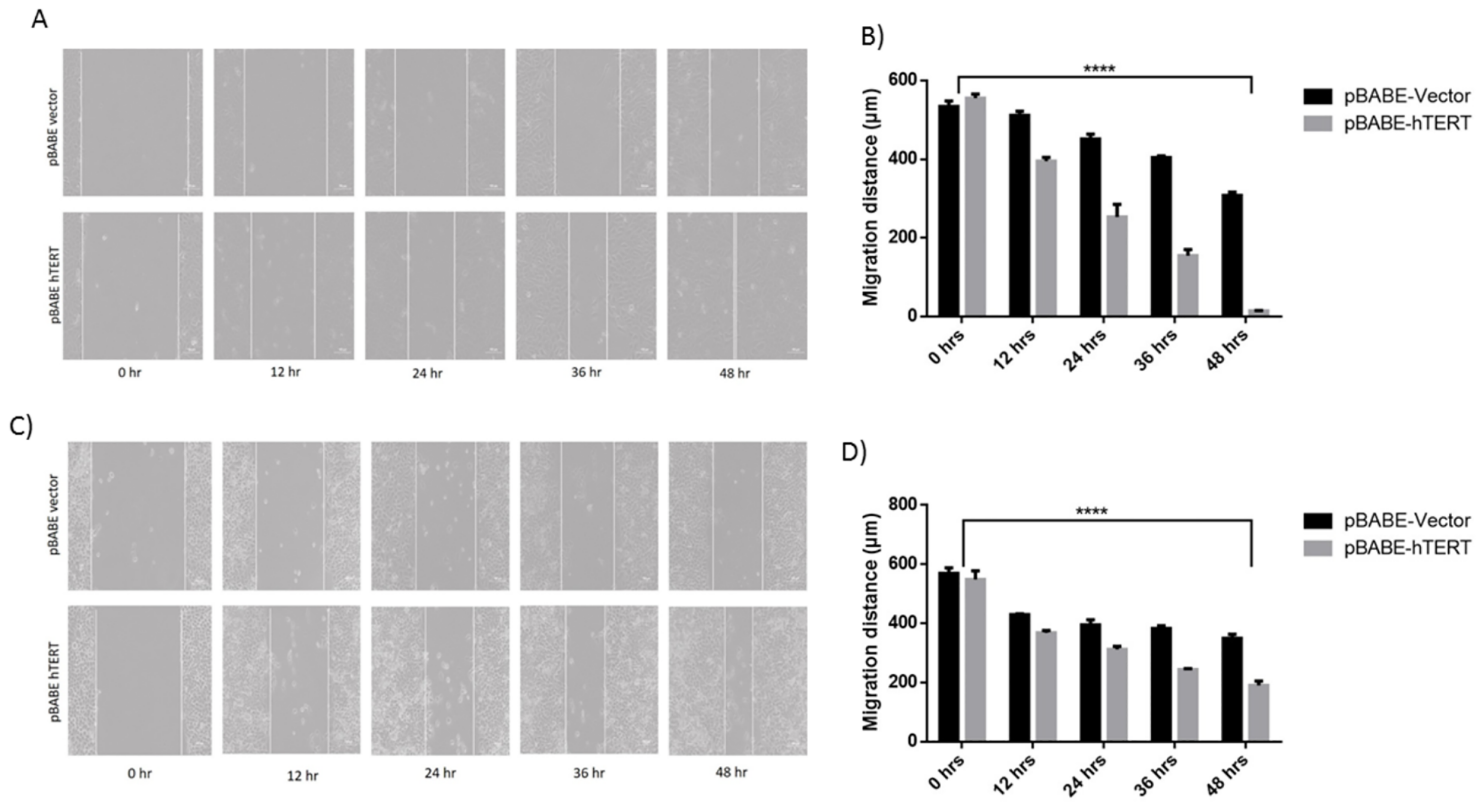
hTERT overexpression enhances the migration of cancer cells. A) Microscopic images of in vitro wound healing at 0, 12, 24, 36 and 48 h after the creation of wounds in U2OS cells. B) Histogram represents quantification of the effect of hTERT overexpression on cell mobility (% migration) in U2OS cell line. C) Microscopic images of in vitro wound healing at 0, 12, 24, 36 and 48 h after the creation of wounds in HeLa cells. D) Histogram representing quantification of the effect of hTERT overexpression on cell mobility (% migration) in HeLa cell line.

The biological consequence of hTERT overexpression in HeLa and U2OS cells was studied employing wound healing assay which revealed increased migration rate of hTERT overexpressing U2OS cells (Fig 2A and 2B) and HeLa cells (Fig 2C and 2D) in comparison to vector transfected cells.

### hTERT overexpression alters the proteomic profile of human cervical cancer and human osteosarcoma cells

**Fig 3:**
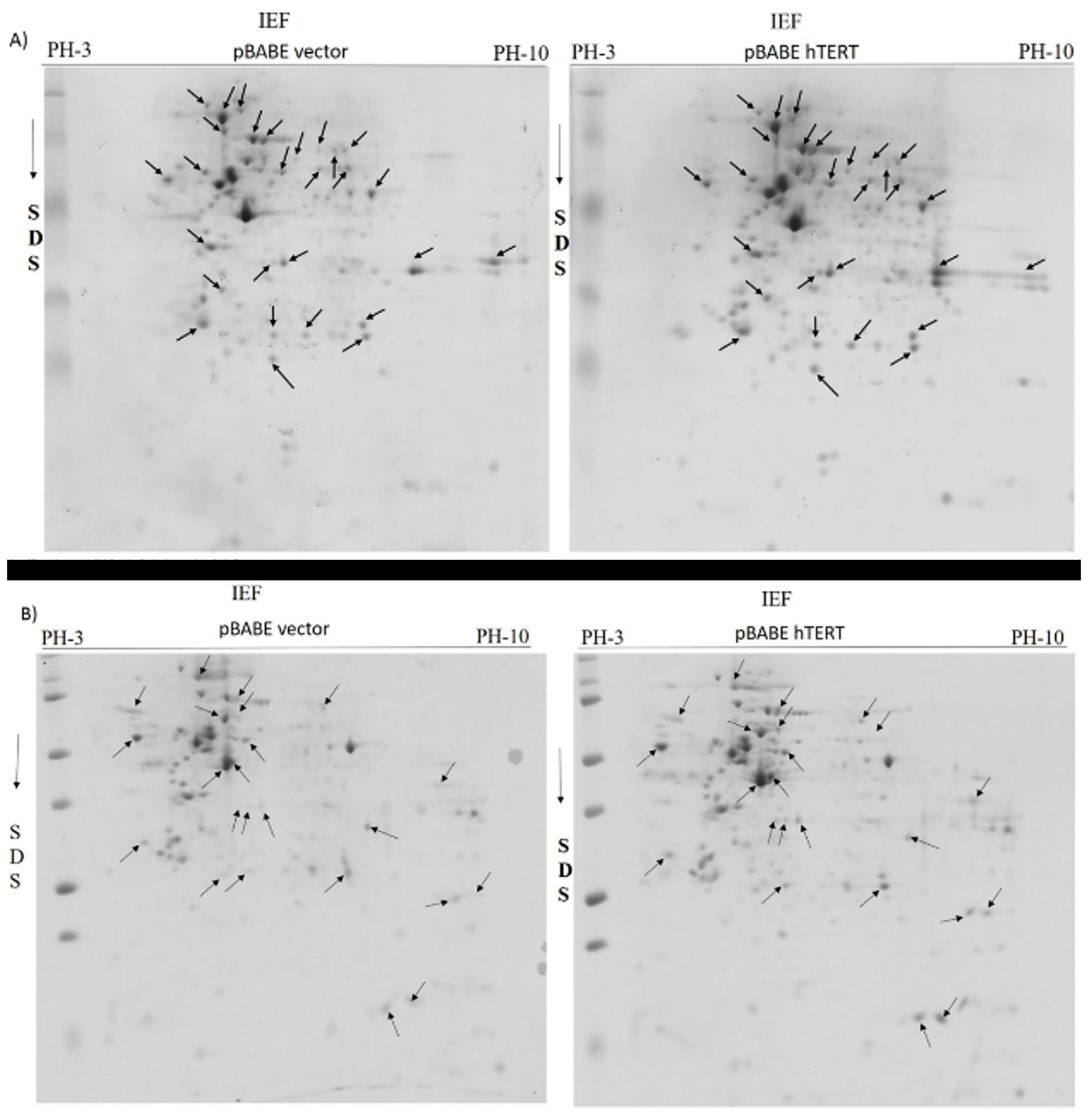
Two dimensional gel electrophoresis of hTERT overexpressing (A) U2OS and (B) HeLa cell line. Total proteins were extracted from hTERT overexpressing U2OS and HeLa cells and separated non-linearly on IPG strip of PH 3–10, followed by electrophoresis through 12% polyacrylamide gels. The gels were further stained and analyzed by image master 2D platinum software.

U2OS is a telomerase negative cell line and it has an ALT pathway to maintain the telomere length and thus it offers a clean baseline for observing any alteration in protein expression after hTERT overexpression in this cell line. After staining and analysis of gels by ImageMaster 2D Platinum v7.0 gel analysis software-(GE Healthcare Life Sciences), 28 spots (Table 2) showed differentially expressed proteins in U2OS cells out of which 23 were upregulated and 5 were down regulated (Fig 3A). In HeLa cells we excised 23 spots (Table 3) representing differentially expressed proteins out of which 21 were up regulated and 2 were downregulated (Fig 3B) proteins. Most of these differentially expressed proteins seemed to be functionally associated with tumorigenesis. We found proteins involved in intermediate filament formation, glycolysis, antioxidant activity, heat shock proteins, apoptosis, nucleotide-sugar biosynthesis, metastasis, xenobiotic metabolism, ubiqutination and glycosylation [Tables 2 and 3]

**Table 2:** List of proteins differentially expressed following hTERT overexpression in U2OS cells.

Spot no.	Protein Identification	Sequence Coverage (%)	pI	Mass (kDa)	Expression level	Score
**1**	Immunoglobulin gamma heavy chain variable region, partial	56.1%	10	109	Downregulation	51
**2**	UDP-glucuronic acid decarboxylase 1 isoform 3	21.8%	9.6	28	Upregulation	47
**3**	Glyceraldehyde-3-phosphate dehydrogenase isoform 2	23.9%	7.9	32	Upregulation	68
**4**	Heat shock protein HSP 90-beta isoform c	21.1%	4.8	83	Upregulation	99
**5**	Heat shock 70kDa protein 8 isoform 1 variant, partial	33.7%	5.2	71	Upregulation	152
**6**	hCG2038865, partial	31.9%	10.3	102	Upregulation	46
**7**	L-lactate dehydrogenase B chain isoform LDHB	11.4%	5.7	37	Upregulation	44
**8**	Unnamed protein product	21.0%	4.6	51	Upregulation	100
**9**	dnaJ homolog subfamily C member 12 isoform X1	30.2%	5.9	137	Upregulation	40
**10**	Alternative protein CDH6	49.4%	11.9	101	Downregulation	43
**11**	Alpha-enolase isoform X1	14.1%	6.7	47	Upregulation	49
**12**	PDZ and LIM domain 2 (mystique), isoform CRA_c, partial	19.9%	13	178	Upregulation	53
**13**	Heat shock 60kDa protein 1 (chaperonin)	12.4%	9.1	60	Upregulation	72
**14**	hCG1789535	15.6%	9.3	50	Downregulation	15
**15**	hCG2040343, partial	55.6%	10.8	79	Upregulation	31
**16**	Enolase 1 variant, partial	21.0%	7.7	47	Upregulation	73
**17**	Mucin, partial	66.7%	12.8	27	Upregulation	38
**18**	Immunoglobulin M heavy chain, partial	100.0%	4.2	28	Upregulation	36
**19**	Glutathione S-transferase P	17.6%	4.1	23	Upregulation	81
**20**	BiP protein, partial	15.2%	5.1	71	downregulation	67
**21**	Ubiquitin carboxy-terminal hydrolase L1, partial	19.2%	5.2	23	downregulation	40
**22**	hCG2045028	35.9%	6	44	Upregulation	36
**23**	Unnamed protein product	10.4%	6.8	26	Upregulation	36
**24**	Unknown, partial	61.5%	5	45	Upregulation	35
**25**	RAMP2	13.7%	5.4	19	Upregulation	41
**26**	Annexin A5	45.6%	4.8	35	Upregulation	155
**27**	T-complex protein 1 subunit beta isoform 1	20.7%	6	58	Upregulation	89
**28**	StAR-related lipid transfer protein 7, mitochondrial precursor	12.4%	9.5	43	Upregulation	41

**Table 3:** List of proteins differentially expressed following hTERT overexpression in HeLa cells.

Spot no.	Protein Identification	Sequence Coverage (%)	pI	Mass (kDa)	Expression level	Score
**1**	Keratin 18	29.3%	5.3	48	Upregulation	68
**2**	Chain A, Crystal Structure Of The Globular Domain Of Human Calreticulin	32.8%	4.6	60	Upregulation	53
**3**	Chain A, Structural Basis Of Human Triosephosphate Isomerase Deficiency. Mutation E104d And Correlation To Solvent Perturbation.	47.2%	6.5	27	Upregulation	88
**4**	Glyceraldehyde-3-phosphate dehydrogenase isoform 2	31.4%	7.9	32	Upregulation	75
**5**	Peroxiredoxin 1, isoform CRA_b, partial	34.6%	6.5	21	Upregulation	47
**6**	Peroxiredoxin-1	62.3%	9.2	22	Upregulation	130
**7**	Chain K, Acetyl-Cypa:cyclosporine Complex	36.4%	7.8	18	Upregulation	73
**8**	Calcium-activated chloride channel regulator family member 3	29.4%	9.2	30	Upregulation	47
**9**	Chain A, Human Heart L-lactate Dehydrogenase H Chain, Ternary Complex With Nadh And Oxamate	27.3%	5.7	37	Upregulation	52
**10**	Mitochondrial ribosomal protein L46, isoform CRA_d	29.5%	9.1	19	Upregulation	34
**11**	F-actin-capping protein subunit alpha-1	33.2%	5.4	33	Upregulation	47
**12**	Chain A, Structural Basis For The Interaction Of Human β-defensin 6 And Its Putative Chemokine Receptor Ccr2 A nd Breast Cancer Microvesicles	59.2%	10.2	57	Upregulation	45
**13**	Heat shock 70kDa protein	14.0%	6	73	Upregulation	69
**14**	Galactose-1-phosphate uridyl transferase	100.0%	9.5	32	Upregulation	40
**15**	T cell receptor alpha, partial	46.5%	9.6	78	Upregulation	40
**16**	PR domain containing 8, isoform CRA_b	39.8%	7.6	19	Upregulation	44
**17**	T cell receptor alpha chain V-J-region, partial	9.6%	9.5	12	Upregulation	39
**18**	Unnamed protein product	19.3%	5.4	59	Upregulation	36
**20**	Chaperonin (HSP60)	29.4%	5.5	60	Upregulation	79
**21**	Chromosome 14 open reading frame 68, isoform CRA_a	60.8%	12.1	78	Upregulation	41
**22**	Cofilin 1 (non-muscle), isoform CRA_c, partial	38.7%	9.4	158	Downregulation	49
**23**	Unnamed protein product	10.4%	6.8	126	Downregulation	36

### Upregulation of Hsp90, Hsp70 and Hsp60

**Fig 4:**
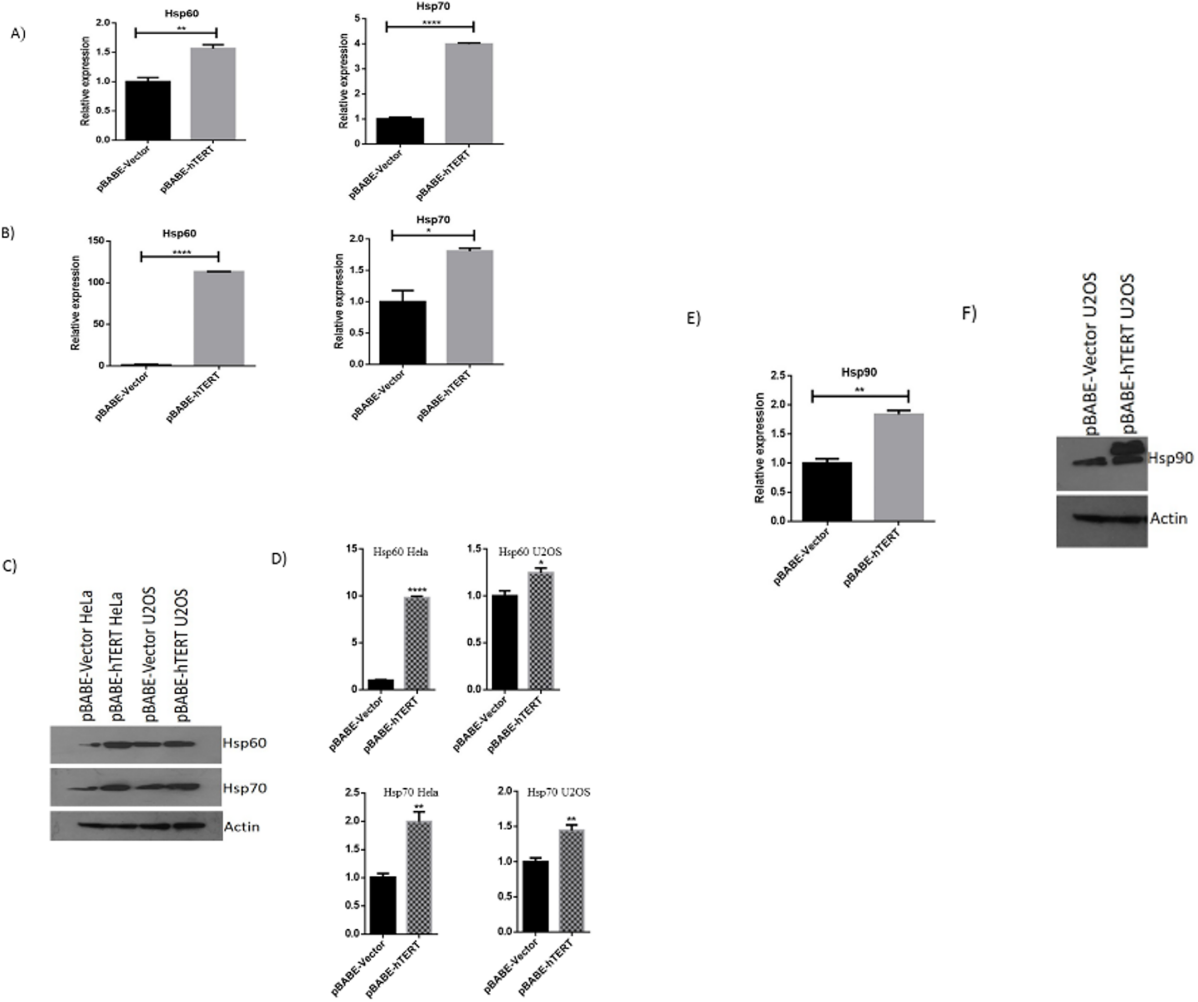
Validation of upregulation of heat shock proteins in U2OS and HeLa cell lines. The hTERT induced upregulation of Hsp60 and hsp70 were confirmed by qRT-PCR in (A) U2OS and (B) HeLa cell lines respectively. C) Western blotting showing upregulation of Hsp60 and Hsp70 at protein level. D) Histogram shows the results by applying ImageJ software. D) To confirm the upregulation of Hsp90 in U2OS cell line E) QRT-PCR and F) western blotting is performed.

We found significant over-expression of heat shock proteins Hsp70 and Hsp60 in hTERT overexpressing U2OS and HeLa cells. Hsp90 was upregulated in only U2OS cells. Hsp90 is an important subunit of telomerase and it helps in stabilizing a functional telomerase structure and in primer loading and extension [13]. Hsp70 is overexpressed in most of the cancer cells though its expression in cancer cells is typically a poor marker for prognosis [14]. Heat shock protein 60 (HSP60) plays a crucial role in malignant cell survival [15]. To confirm regulation of Hsp60 and Hsp70 by hTERT, we performed qRT-PCR (Fig 4A and 4B) and western blotting (Fig 4C & 4D) to check their expression in U2OS and HeLa cells carrying pBABE-Vector and pBABE-hTERT expression construct. QRT-PCR and Western blotting confirmed the upregulation of Hsp90 at transcript as well as protein level in U2OS cells (Fig 4E).

### Upregulation of GAPDH

**Fig 5:**
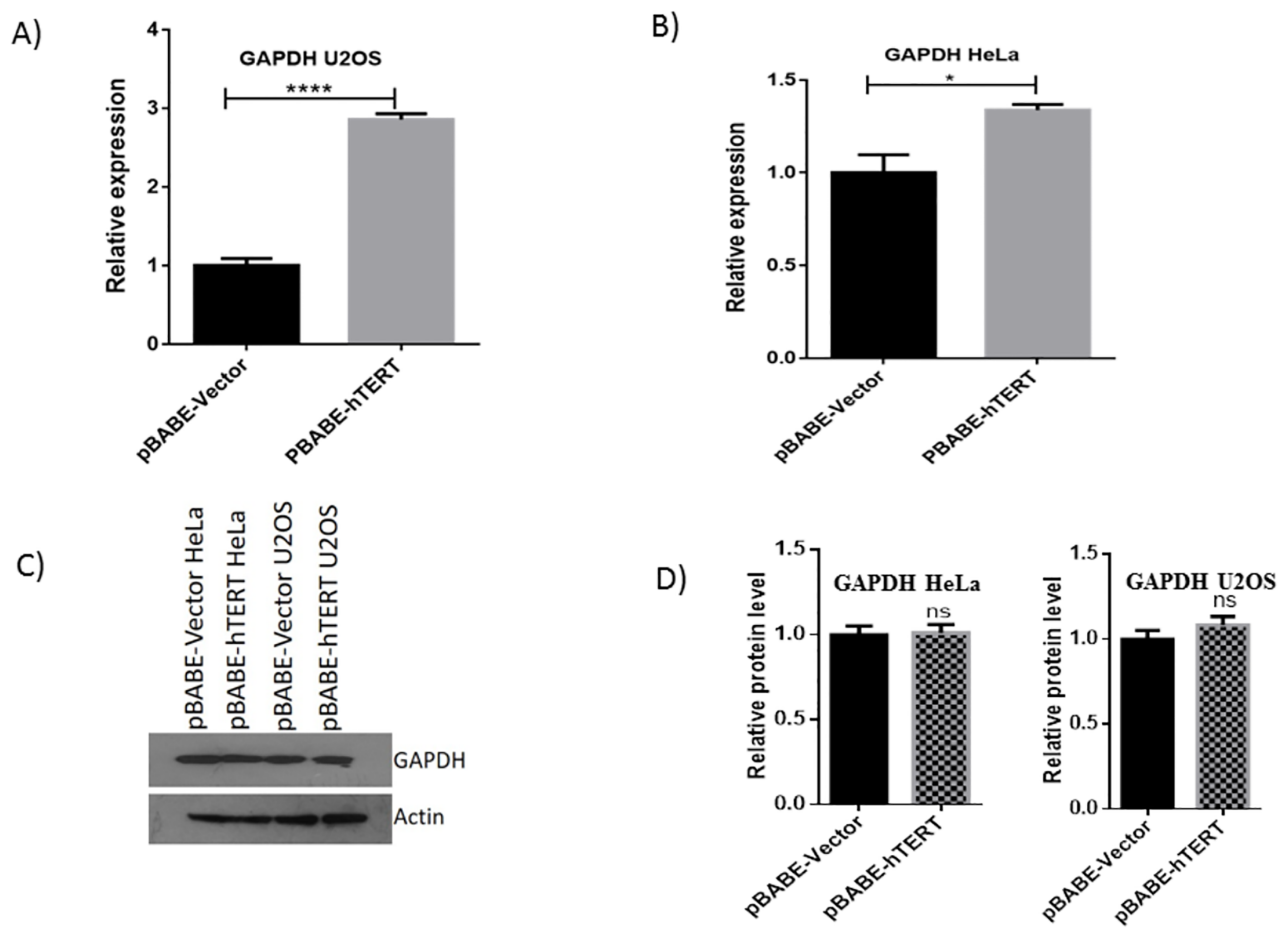
Validation of GAPDH in HeLa and U2OS cell lines. QRT-PCR was performed to check the hTERT induced upregulation of GAPDH in (A) U2OS and (B) HeLa cell lines. C) Represents results of Western blotting to check the up-regulation of GAPDH at protein level while (D) represents histograms of respective blots showing no prominent difference in expression of GAPDH at protein level.

Glyceraldehyde-3-phosphate dehydrogenase (GAPDH) is basically a glycolytic enzyme and a well-known housekeeping marker and commonly used as an endogenous control to assess cancer related gene expression. However, reports indicate implication of GAPDH in other diverse functions independently of its role in energy metabolism. Deregulation in the expression level of GAPDH are found in many cancer cells [16]. Expression of GAPDH was enhanced in both U2OS and HeLa cells overexpressing hTERT. To confirm the upregulation of GAPDH by hTERT we performed qRT-PCR (5A & 5B) and western blotting (Fig 5C and 5D) in both U2OS and HeLa cell line. We found hTERT causes upregulation of GAPDH at transcriptional level only, there being no conspicuous change at protein level in either cell line.

## Discussion

Telomerase components perform many functions apart from its canonical role in telomere lengthening. One such extracurricular function of hTERT is the regulation of cell’s signaling pathways. Many types of cancer do not express telomerase and being telomerase negative cannot manifest extracurricular activity of telomerase. They have ALT pathway to maintain telomere length. Remarkably, hTERT could be expressed at very high levels even in cells with a basal endogenous level of expression and allowed normal viability. Telomerase negative cells nevertheless offer a good experimental system to study the effect of hTERT overexpression on expression of other genes. Human osteosarcoma is a primary malignant tumor of the bone and the U2OS cell line derived from it is telomerase negative. In the present study, we document, for the first time significant effect of hTERT overexpression on the proteomic profile of U2OS cells. Here, we found Hsp90, Hsp70 and Hsp60 upregulated after hTERT overexpression in this cell line. Heat shock proteins usually act as molecular chaperones and are expressed at high levels in many cancers, although Hsp overexpression is only a poor prognosis in terms of survival and response to therapy in specific cancer types [17]. Heat shock protein 90 (Hsp90) is an abundant molecular chaperone that helps in conformational maturation and stabilization of various oncogenic proteins leading to tumor cell survival and disease progression [18]. Hsp90 interacts with a variety of intracellular proteins and is involved in differentiation, survival and cell growth [13]. It is demonstrated that inhibiting Hsp90 in osteosarcoma cells induces apoptosis [18]. Jennifer McCleese (2009) reported that inhibitioin of Hsp90 results in loss of cell viability, induction of apoptosis, and inhibition of cell proliferation in osteosarcoma cells [19]. It has also been shown that blocking HSP90 addiction inhibits tumor cell proliferation, metastasis and development [20]. Moreover, Hsp90 is also a subunit of telomerase complex and it stabilizes telomerase and helps in loading of telomerase complex to telomere [13]. Though there is no hTERT in U2OS cells, its overexpression upregulated Hsp90 (Fig 4d) suggesting intactness of the circuitry of intercommunication between hTERT and Hsps in telomerase negative cells.

Similar to Hsp90, the role of Hsp70 in cancer is very well studied. Being a molecular chaperone, Hsp70 is an important part of cellular networks, involving signaling, membrane, transcriptional and organelles functions [21]. In many cancer increased expression of Hsp70 is correlated with poor prognosis [21]. Lei Zhao et al. reported that viability of osteosarcoma cells was adversely affected after knocking down of Hsp70 [22]. It is also demonstrated that Hsp70 expression prevents apoptosis in osteosarcoma cells [23]. The role of Hsp70 in invasion and metastasis of cancer cells are also very well studied [24]. Moreover, Hsp70 inhibitor in cervical cancer also inhibits cancer cell proliferation [25]. R Ralhan et al. (1995) found that, Hsp70 overexpression can be correlated with elevated proliferation and tumor size in uterine cervical cancer [26]. Importantly, Hsp70 is a potent buffering system for cellular stress either from extrinsic (physiological, viral and environmental) or intrinsic (replicative or oncogenic) stimuli. For survival, cancer cells depend heavily on this buffering system [14]. Moreover, reports have also shown association of hTERT with Hsp70. Hsp70 binds with hTERT when hTR is absent and it gets dissociated when telomerase is folded into its active state [27] suggesting a transient association of Hsp70 with telomerase. We found upregulation of Hsp70 both at RNA level and protein level (Fig 4A & 4C). Another heat shock protein showing differential expression in association with hTERT is Hsp60 which has been reported to interact with hTERT in mitochondria [28]. Initially, Hsp60 was found only in mitochondria but since last few year, studies confirmed its presence the cytosol, the cell surface and in the extracellular space [29]. Inside the mitochondria it was reported that it binds mainly with Hsp10. However, in addition to its association with Hsp10, other interacting molecules have also been identified for Hsp60 in recent years [29]. Similar to Hsp70, the role of Hsp60 in cancer proliferation, tumor cell survival and metastasis are very well demonstrated in both osteosarcoma and cervical cancer cells [30,31].

We also found upregulation of Hsp60 and Hsp70 in HeLa cells which, unlike U2OS, are telomerase positive (Fig 4B & 4C). In the present study we have determined that hTERT may be an important modulator of heat shock proteins in these cells. However, it will be necessary to scan a wider range of cells for their responses in order to arrive at any generalization.

Yet another protein which was differentially overexpressed is GAPDH which is product of a housekeeping gene and is commonly used as internal control in different experimental condition. This enzyme is mainly used during glycolysis but it also has many diverse functions independent of its role in glycolysis [32]. Recent findings including our own show that expression level of GAPDH is highly regulated in various cancer cells [16].

There are signs of involvement of GAPDH in cancer progression and it may serve as a new marker or even a therapeutic target [16]. Moreover, it has been already shown that GAPDH mediates many oxidative stress responses, including nuclear translocation of GAPDH and induction of cell death. Craig Nicholls et al.(2012) reported that GAPDH interacts with telomerase RNA component hTR and inhibits telomerase activity which leads to telomere shortening and senescence in breast cancer cells [33]. Here we show that hTERT overexpression leads to the upregulation of GAPDH in both U2OS and HeLa cell lines suggesting that GAPDH may also interact with hTERT but in a positive manner. We do not find any cell death after hTERT overexpression. Apart from heat shock proteins and GAPDH there are many other proteins found to be differentially regulated. Keratin 18 is an intermediate filament protein and can be used for detection of proliferating fractions in the breast cancer [34]. This protein is found upregulated in HeLa cells following hTERT overexpression suggesting that hTERT modulates the expression of cytoskeletal proteins in cancer cells. Another upregulated protein we found is peroxiredoxin 1. This is an antioxidant enzyme associates with telomeres and protects it from oxidative damage and preserves telomeres for extension by telomerase [35]. Upregulation of peroxiredoxin in hTERT overexpressing HeLa cells indicates that hTERT enhances the expression of peroxiredoxin 1 to protect telomeres from any oxidative damage. It is well known that hTERT promotes EMT and during EMT there is change in expression of some epithelial and mesenchymal markers; level of mesenchymal markers goes up while that of epithelial markers goes down. CDH6 which is a type 2 cadherin and an epithelial marker drives EMT during embryonic development and it is aberrantly re-activated in cancer [36]. We found downregulation of CDH6 in U2OS cells when hTERT is overexpressed showing that hTERT promotes mesenchymal character in these cells. Another down regulated protein we found is ubiquitin carboxyl-terminal hydrolase L1 (UCHL1). It is a cysteine protease belongs to the UCH proteases family and has also acquired E3 ubiquitin-protein ligase activity and stabilizes ubiquitin monomers in vivo [37]. This protein has heterogeneous expression in cancer cells and performs both tumor inhibition and promoting functions [38]. Down regulation of this protein in U2OS cells after hTERT overexpression indicates that hTERT modulates the expression of this deubiquitinating enzyme to avoid proteosomal degradation of itself. Furthermore, apart from GAPDH another glycolytic enzyme upregulated is alpha-enolase which is now used as a potential cancer prognostic marker and promotes invasion in cancer cells [39] indicating promotion of invasion by hTERT via regulation of these glycolytic enzymes. We also found that hTERT expression led to the increase in migration rate of both U2OS and HeLa cells indicating that higher level of hTERT is linked with high invasive tumor phenotype.

In conclusion, this study shows that hTERT expression alters the proteomic profile of osteosarcoma and cervical cancer cells. Moreover, heat shock proteins are an important subset of cellular proteins regulated by hTERT. GAPDH is also influenced by hTERT expression in both the cell lines. The findings make it pertinent to further investigate the relevance of telomerase associated molecules like peroxiredoxin-1 and alpha-enolase, as markers or therapeutic target.

## Supporting information

S1 File Table A: Real time data of hTERT overexpression in U2OS cells. Fig A: Western blotting of hTERT overexpression in U2OS cells. Table B: Densitometric quantification of western blot. Table C: Real time data of hTERT overexpression in HeLa cells. Fig B: Western blotting of hTERT overexpression in HeLa cells. Table D: Densitometric quantification of western blot. Fig C: Wound healing assay in U2OS cells. Table E: Table 5: Distance migrated (μm) value which is used to make graph in hTERT overexpressing U2OS cells. Fig D: Wound healing assay in HeLa cells. Table F: Distance migrated (μm) value which is used to make graph in hTERT overexpressing HeLa cells. Fig E: Two dimensional gel electrophoresis of vector expressed U2OS cells. Fig F: Two dimensional gel electrophoresis of hTERT overexpressing U2OS cells. Fig G: Two dimensional gel electrophoresis of vector expressed HeLa cells. Fig H: Two dimensional gel electrophoresis of hTERT overexpressing HeLa cells. Table G: Real-time data of Hsp60 in U2OS cells. Table H: Real time data of Hsp70 in U2OS cells. Table I: Real-time data of Hsp60 in HeLa cells. Table J: Real time data of Hsp70 in HeLa cells. Fig I: Western blotting of Hsp60 and Hsp70 in U2OS and HeLa cells following hTERT overexpression. Table K: Distance migrated (μm) value which is used to make graph of Hsp60 in HeLa cells. Table L: Distance migrated (μm) value which is used to make graph of Hsp60 in U2OS cells. Table M: Distance migrated (μm) value which is used to make graph of Hsp70 in HeLa cells. Table N: Distance migrated (μm) value which is used to make graph of Hsp70 in U2OS cells. Table O: Real time data of Hsp90 in U2OS cells. Fig J: Western blotting of Hsp90 in U2OS cells following hTERT overexpression. Table P: Real time data of GAPDH in U2OS cells. Table Q: Real time data of GAPDH in HeLa cells. Fig K: Western blotting of GAPDH in U2OS and HeLa cells following hTERT overexpression. Table R: Densitometric quantification of GAPDH in U2OS cells. Table S: Densitometric quantification of GAPDH in HeLa cells.
(DOC)

## References

[1] Chang JT-C, Chen Y-L, Yang H-T, Chen C-Y, Cheng A-J. Differential regulation of telomerase activity by six telomerase subunits. Eur J Biochem. 2002;269: 3442–3450. 12135483 10.1046/j.1432-1033.2002.03025.x

[2] Jaiswal RK, Kumar P, Yadava PK. Telomerase and its extracurricular activities. Cell Mol Biol Lett. 2013;18: 538–554. doi: 10.2478/s11658-013-0105-0 24048710 10.2478/s11658-013-0105-0 PMC6275585

[3] Bodnar AG, Ouellette M, Frolkis M, Holt SE, Chiu CP, Morin GB, et al Extension of life-span by introduction of telomerase into normal human cells. Science. 1998;279: 349–352. 9454332 10.1126/science.279.5349.349

[4] Flores I, Benetti R, Blasco MA. Telomerase regulation and stem cell behaviour. Curr Opin Cell Biol. 2006;18: 254–260. doi: 10.1016/j.ceb.2006.03.003 16617011 10.1016/j.ceb.2006.03.003

[5] Tsai C-C, Chen C-L, Liu H-C, Lee Y-T, Wang H-W, Hou L-T, et al Overexpression of hTERT increases stem-like properties and decreases spontaneous differentiation in human mesenchymal stem cell lines. J Biomed Sci. 2010;17: 64 doi: 10.1186/1423-0127-17-64 20670406 10.1186/1423-0127-17-64 PMC2923118

[6] Zhang X, Mar V, Zhou W, Harrington L, Robinson MO. Telomere shortening and apoptosis in telomerase-inhibited human tumor cells. Genes Dev. 1999;13: 2388–2399. 10500096 10.1101/gad.13.18.2388 PMC317024

[7] Mazzucchelli GD, Gabelica V, Smargiasso N, Fléron M, Ashimwe W, Rosu F, et al Proteome alteration induced by hTERT transfection of human fibroblast cells. Proteome Sci. 2008;6: 12 doi: 10.1186/1477-5956-6-12 18419814 10.1186/1477-5956-6-12 PMC2386453

[8] Uziel O, Yosef N, Sharan R, Ruppin E, Kupiec M, Kushnir M, et al The effects of telomere shortening on cancer cells: a network model of proteomic and microRNA analysis. Genomics. 2015;105: 5–16. doi: 10.1016/j.ygeno.2014.10.013 25451739 10.1016/j.ygeno.2014.10.013

[9] Diao S, Zhang J, Wang H, He M, Lin MC, Chen Y, et al Proteomic identification of microRNA-122a target proteins in hepatocellular carcinoma. Proteomics. 2010;10: 3723–3731. doi: 10.1002/pmic.201000050 20859956 10.1002/pmic.201000050

[10] Dyballa N, Metzger S. Fast and sensitive colloidal coomassie G-250 staining for proteins in polyacrylamide gels. J Vis Exp JoVE. 2009; doi: 10.3791/1431 19684561 10.3791/1431 PMC3149902

[11] Shevchenko A, Wilm M, Vorm O, Mann M. Mass spectrometric sequencing of proteins silver-stained polyacrylamide gels. Anal Chem. 1996;68: 850–858. 8779443 10.1021/ac950914h

[12] Cai J, Tang H, Xu L, Wang X, Yang C, Ruan S, et al Fibroblasts in omentum activated by tumor cells promote ovarian cancer growth, adhesion and invasiveness. Carcinogenesis. 2012;33: 20–29. doi: 10.1093/carcin/bgr230 22021907 10.1093/carcin/bgr230

[13] Keppler BR, Grady AT, Jarstfer MB. The biochemical role of the heat shock protein 90 chaperone complex in establishing human telomerase activity. J Biol Chem. 2006;281: 19840–19848. doi: 10.1074/jbc.M511067200 16714764 10.1074/jbc.M511067200

[14] Murphy ME. The HSP70 family and cancer. Carcinogenesis. 2013;34: 1181–1188. doi: 10.1093/carcin/bgt111 23563090 10.1093/carcin/bgt111 PMC3670260

[15] Hjerpe E, Egyhazi S, Carlson J, Stolt MF, Schedvins K, Johansson H, et al HSP60 predicts survival in advanced serous ovarian cancer. Int J Gynecol Cancer Off J Int Gynecol Cancer Soc. 2013;23: 448–455. doi: 10.1097/IGC.0b013e318284308b 23429486 10.1097/IGC.0b013e318284308b

[16] Zhang J-Y, Zhang F, Hong C-Q, Giuliano AE, Cui X-J, Zhou G-J, et al Critical protein GAPDH and its regulatory mechanisms in cancer cells. Cancer Biol Med. 2015;12: 10–22. doi: 10.7497/j.issn.2095-3941.2014.0019 25859407 10.7497/j.issn.2095-3941.2014.0019 PMC4383849

[17] van de Vijver MJ, He YD, van’t Veer LJ, Dai H, Hart AAM, Voskuil DW, et al A gene-expression signature as a predictor of survival in breast cancer. N Engl J Med. 2002;347: 1999–2009. doi: 10.1056/NEJMoa021967 12490681 10.1056/NEJMoa021967

[18] Mori M, Hitora T, Nakamura O, Yamagami Y, Horie R, Nishimura H, et al Hsp90 inhibitor induces autophagy and apoptosis in osteosarcoma cells. Int J Oncol. 2015;46: 47–54. doi: 10.3892/ijo.2014.2727 25351442 10.3892/ijo.2014.2727 PMC4238730

[19] McCleese JK, Bear MD, Fossey SL, Mihalek RM, Foley KP, Ying W, et al The novel HSP90 inhibitor STA-1474 exhibits biologic activity against osteosarcoma cell lines. Int J Cancer. 2009;125: 2792–2801. doi: 10.1002/ijc.24660 19544563 10.1002/ijc.24660

[20] Ory B, Baud’huin M, Verrecchia F, Royer BB-L, Quillard T, Amiaud J, et al Blocking HSP90 Addiction Inhibits Tumor Cell Proliferation, Metastasis Development, and Synergistically Acts with Zoledronic Acid to Delay Osteosarcoma Progression. Clin Cancer Res Off J Am Assoc Cancer Res. 2016;22: 2520–2533. doi: 10.1158/1078-0432.CCR-15-1925 26712686 10.1158/1078-0432.CCR-15-1925

[21] Soti C, Pál C, Papp B, Csermely P. Molecular chaperones as regulatory elements of cellular networks. Curr Opin Cell Biol. 2005;17: 210–215. doi: 10.1016/j.ceb.2005.02.012 15780599 10.1016/j.ceb.2005.02.012

[22] Zhao L, Jiang B, Wang D, Liu W, Zhang H, Liu W, et al Triptolide reduces the viability of osteosarcoma cells by reducing MKP-1 and Hsp70 expression. Exp Ther Med. 2016;11: 2005–2010. doi: 10.3892/etm.2016.3164 27168842 10.3892/etm.2016.3164 PMC4840823

[23] Ding L, He S, Sun X. HSP70 desensitizes osteosarcoma cells to baicalein and protects cells from undergoing apoptosis. Apoptosis Int J Program Cell Death. 2014;19: 1269–1280. doi: 10.1007/s10495-014-0995-y 24846187 10.1007/s10495-014-0995-y

[24] Juhasz K, Lipp A-M, Nimmervoll B, Sonnleitner A, Hesse J, Haselgruebler T, et al The complex function of hsp70 in metastatic cancer. Cancers. 2013;6: 42–66. doi: 10.3390/cancers6010042 24362507 10.3390/cancers6010042 PMC3980608

[25] Liu J, Liu J, Guo S-Y, Liu H-L, Li S-Z. HSP70 inhibitor combined with cisplatin suppresses the cervical cancer proliferation in vitro and transplanted tumor growth: An experimental study. Asian Pac J Trop Med. 2017;10: 184–188. doi: 10.1016/j.apjtm.2017.01.020 28237487 10.1016/j.apjtm.2017.01.020

[26] Ralhan R, Kaur J. Differential expression of Mr 70,000 heat shock protein in normal, premalignant, and malignant human uterine cervix. Clin Cancer Res. 1995;1: 1217–1222. 9815915

[27] Forsythe HL, Jarvis JL, Turner JW, Elmore LW, Holt SE. Stable association of hsp90 and p23, but Not hsp70, with active human telomerase. J Biol Chem. 2001;276: 15571–15574. doi: 10.1074/jbc.C100055200 11274138 10.1074/jbc.C100055200

[28] Sharma NK, Reyes A, Green P, Caron MJ, Bonini MG, Gordon DM, et al Human telomerase acts as a hTR-independent reverse transcriptase in mitochondria. Nucleic Acids Res. 2012;40: 712–725. doi: 10.1093/nar/gkr758 21937513 10.1093/nar/gkr758 PMC3258147

[29] Cappello F, Conway de Macario E, Marasà L, Zummo G, Macario AJL. Hsp60 expression, new locations, functions and perspectives for cancer diagnosis and therapy. Cancer Biol Ther. 2008;7: 801–809. 18497565 10.4161/cbt.7.6.6281

[30] Selvarajah GT, Bonestroo FAS, Kirpensteijn J, Kik MJL, van der Zee R, van Eden W, et al Heat shock protein expression analysis in canine osteosarcoma reveals HSP60 as a potentially relevant therapeutic target. Cell Stress Chaperones. 2013;18: 607–622. doi: 10.1007/s12192-013-0414-2 23463150 10.1007/s12192-013-0414-2 PMC3745254

[31] Tsai Y-P, Yang M-H, Huang C-H, Chang S-Y, Chen P-M, Liu C-J, et al Interaction between HSP60 and beta-catenin promotes metastasis. Carcinogenesis. 2009;30: 1049–1057. doi: 10.1093/carcin/bgp087 19369584 10.1093/carcin/bgp087

[32] Sirover MA. New insights into an old protein: the functional diversity of mammalian glyceraldehyde-3-phosphate dehydrogenase. Biochim Biophys Acta. 1999;1432: 159–184. 10407139 10.1016/s0167-4838(99)00119-3

[33] Nicholls C, Pinto AR, Li H, Li L, Wang L, Simpson R, et al Glyceraldehyde-3-phosphate dehydrogenase (GAPDH) induces cancer cell senescence by interacting with telomerase RNA component. Proc Natl Acad Sci U S A. 2012;109: 13308–13313. doi: 10.1073/pnas.1206672109 22847419 10.1073/pnas.1206672109 PMC3421169

[34] Ha S-A, Lee YS, Kim HK, Yoo J, Kim S, Gong G-H, et al The prognostic potential of keratin 18 in breast cancer associated with tumor dedifferentiation, and the loss of estrogen and progesterone receptors. Cancer Biomark Sect Dis Markers. 2011;10: 219–231. doi: 10.3233/CBM-2012-0250 22699783 10.3233/CBM-2012-0250 PMC13016247

[35] Aeby E, Ahmed W, Redon S, Simanis V, Lingner J. Peroxiredoxin 1 Protects Telomeres from Oxidative Damage and Preserves Telomeric DNA for Extension by Telomerase. Cell Rep. 2016;17: 3107–3114. doi: 10.1016/j.celrep.2016.11.071 28009281 10.1016/j.celrep.2016.11.071

[36] Gugnoni M, Sancisi V, Gandolfi G, Manzotti G, Ragazzi M, Giordano D, et al Cadherin-6 promotes EMT and cancer metastasis by restraining autophagy. Oncogene. 2017;36: 667–677. doi: 10.1038/onc.2016.237 27375021 10.1038/onc.2016.237

[37] Hurst-Kennedy J, Chin L-S, Li L. Ubiquitin C-terminal hydrolase l1 in tumorigenesis. Biochem Res Int. 2012;2012: 123706 doi: 10.1155/2012/123706 22811913 10.1155/2012/123706 PMC3395355

[38] Wulfänger J, Biehl K, Tetzner A, Wild P, Ikenberg K, Meyer S, et al Heterogeneous expression and functional relevance of the ubiquitin carboxyl-terminal hydrolase L1 in melanoma. Int J Cancer. 2013;133: 2522–2532. doi: 10.1002/ijc.28278 23686552 10.1002/ijc.28278

[39] Song Y, Luo Q, Long H, Hu Z, Que T, Zhang X ‘an, et al Alpha-enolase as a potential cancer prognostic marker promotes cell growth, migration, and invasion in glioma. Mol Cancer. 2014;13: 65 doi: 10.1186/1476-4598-13-65 24650096 10.1186/1476-4598-13-65 PMC3994408

