# Supplementary material for: Proteomic identification of proteins differentially expressed following overexpression of hTERT (human telomerase reverse transcriptase) in cancer cells
Source: PLoS One. 2017 Jul 13;12(7):e0181027. doi: 10.1371/journal.pone.0181027 (PMC5509255; doi:10.1371/journal.pone.0181027)
Supplement: S1 File — Table A: Real time data of hTERT overexpression in U2OS cells. Fig A: Western blotting of hTERT overexpression in U2OS cells. Table B: Densitometric quantification of western blot. Table C: Real time data of hTERT overexpression in HeLa cells. Fig B: Western blotting of hTERT overexpression in HeLa cells. Table D: Densitometric quantification of western blot. Fig C: Wound healing assay in U2OS cells. Table E: Table 5: Distance migrated (μm) value which is used to make graph in hTERT overexpressing U2OS cells. Fig D: Wound healing assay in HeLa cells. Table F: Distance migrated (μm) value which is used to make graph in hTERT overexpressing HeLa cells. Fig E: Two dimensional gel electrophoresis of vector expressed U2OS cells. Fig F: Two dimensional gel electrophoresis of hTERT overexpressing U2OS cells. Fig G: Two dimensional gel electrophoresis of vector expressed HeLa cells. Fig H: Two dimensional gel electrophoresis of hTERT overexpressing HeLa cells. Table G: Real-time data of Hsp60 in U2OS cells. Table H: Real time data of Hsp70 in U2OS cells. Table I: Real-time data of Hsp60 in HeLa cells. Table J: Real time data of Hsp70 in HeLa cells. Fig I: Western blotting of Hsp60 and Hsp70 in U2OS and HeLa cells following hTERT overexpression. Table K: Distance migrated (μm) value which is used to make graph of Hsp60 in HeLa cells. Table L: Distance migrated (μm) value which is used to make graph of Hsp60 in U2OS cells. Table M: Distance migrated (μm) value which is used to make graph of Hsp70 in HeLa cells. Table N: Distance migrated (μm) value which is used to make graph of Hsp70 in U2OS cells. Table O: Real time data of Hsp90 in U2OS cells. Fig J: Western blotting of Hsp90 in U2OS cells following hTERT overexpression. Table P: Real time data of GAPDH in U2OS cells. Table Q: Real time data of GAPDH in HeLa cells. Fig K: Western blotting of GAPDH in U2OS and HeLa cells following hTERT overexpression. Table R: Densitometric quantification of GAPDH in U2OS cells. Table S: D [file pone.0181027.s001.doc]

Supporting information file 1

Table A: Real time data of hTERT overexpression in U2OS cells. Real-time PCR was performed to check overexpression of hTERT in U2OS cells. Fold change values are given in the table.

| Name | Fold Change Value | ΔCт SE |
| --- | --- | --- |
| pBABE Vector U2OS | 1 | 0.400299 |
| pBABE hTERT U2OS | 186.7675 | 0.095716 |


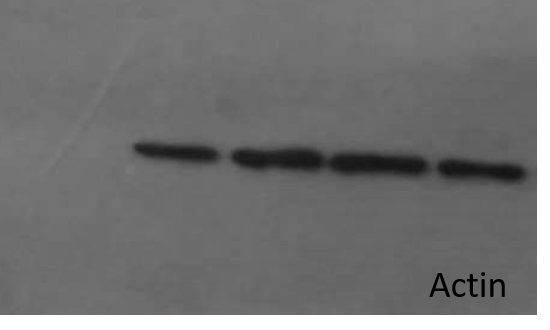

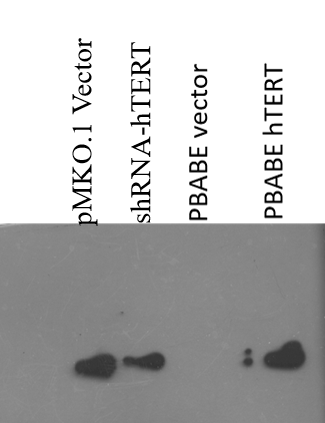
Fig A: Western blotting of hTERT overexpression in U2OS cells.

Fig A: Western blotting is performed to confirm hTERT overexpression in U2OS cells.

Table B: Densitometric quantification of western blot. ImageJ software was used to calculate change in the expression of protein. Fold change values are given in the table.

| Name | Fold Change Value | Standard error of the mean |
| --- | --- | --- |
| pBABE Vector U2OS | 1 | 0.0834 |
| pBABE hTERT U2OS | 5.743 | 0.0654 |

Table C: Real time data of hTERT overexpression in HeLa cells. Real-time PCR was performed to check overexpression of hTERT in HeLa cells. Fold change values are given in the table.

| Name | Fold Change Value | ΔCт SE |
| --- | --- | --- |
| pBABE Vector U2OS | 1 | 0.048267 |
| pBABE hTERT U2OS | 35.38653 | 0.139579 |

Fig B: Western blotting of hTERT overexpression in HeLa cells.


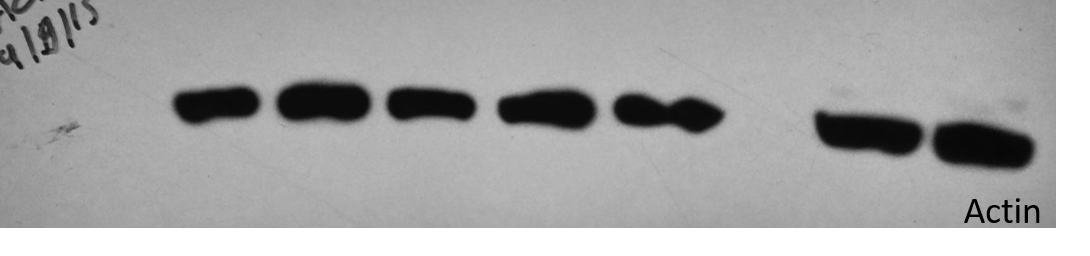

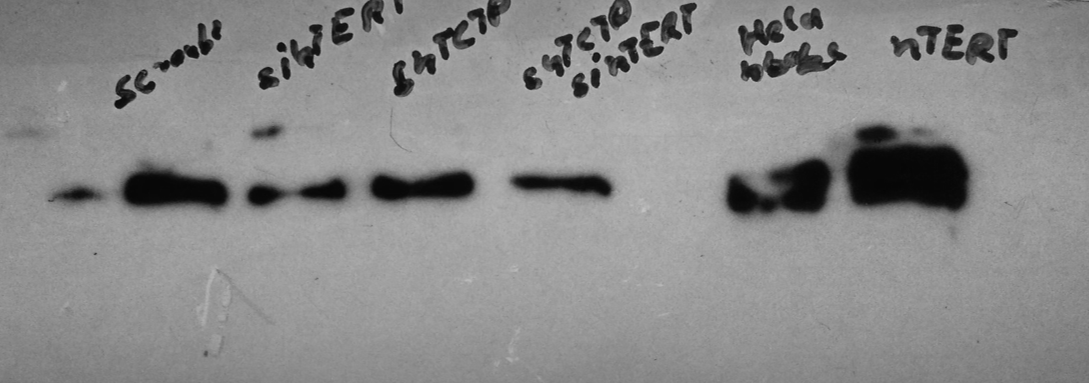


Fig B: Western blotting is performed to confirm hTERT overexpression in U2OS cells.

Table D: Densitometric quantification of western blot. ImageJ software was used to calculate change in the expression of protein. Fold change values are given in the table.

| Name | Fold Change Value | Standard error of the mean |
| --- | --- | --- |
| pBABE Vector HeLa | 1 | 0.1143 |
| pBABE hTERT HeLa | 2.9312 | 0.0943 |

Fig C: Wound healing assay in U2OS cells-
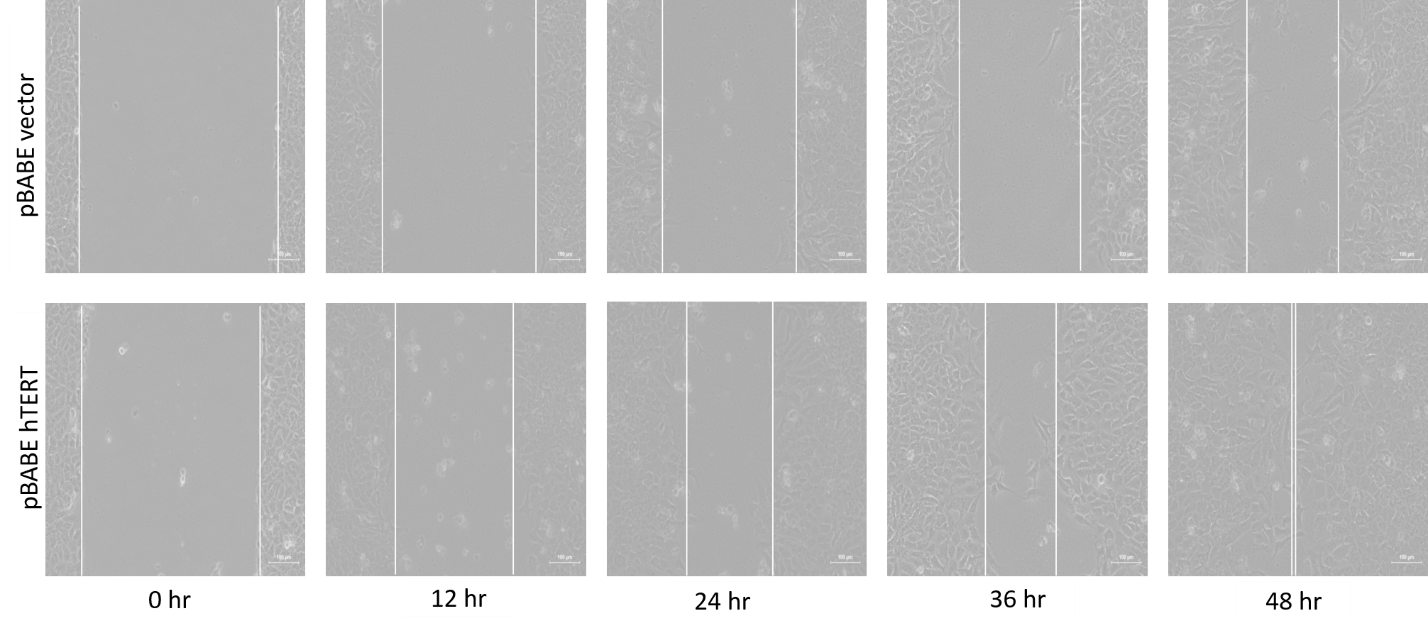


Fig C: Wound healing assay was performed in hTERT overexpressing U2OS cells.

Table E: Distance migrated (µm) value which is used to make graph in hTERT overexpressing U2OS cells. Wound healing assay was performed in hTERT overexpressing U2OS cells. Calculated distance are given in the table.

| Time | pBABE Vector | pBABE hTERT |
| --- | --- | --- |
| 0 hr | 534 | 566 |
| 12hr | 500 | 406 |
| 24 hr | 441 | 224 |
| 36 hr | 404 | 167 |
| 48 hr | 300 | 13 |

|  |
| --- |
|  |
|  |
|  |
|  |

Fig D: Wound healing assay in HeLa cells


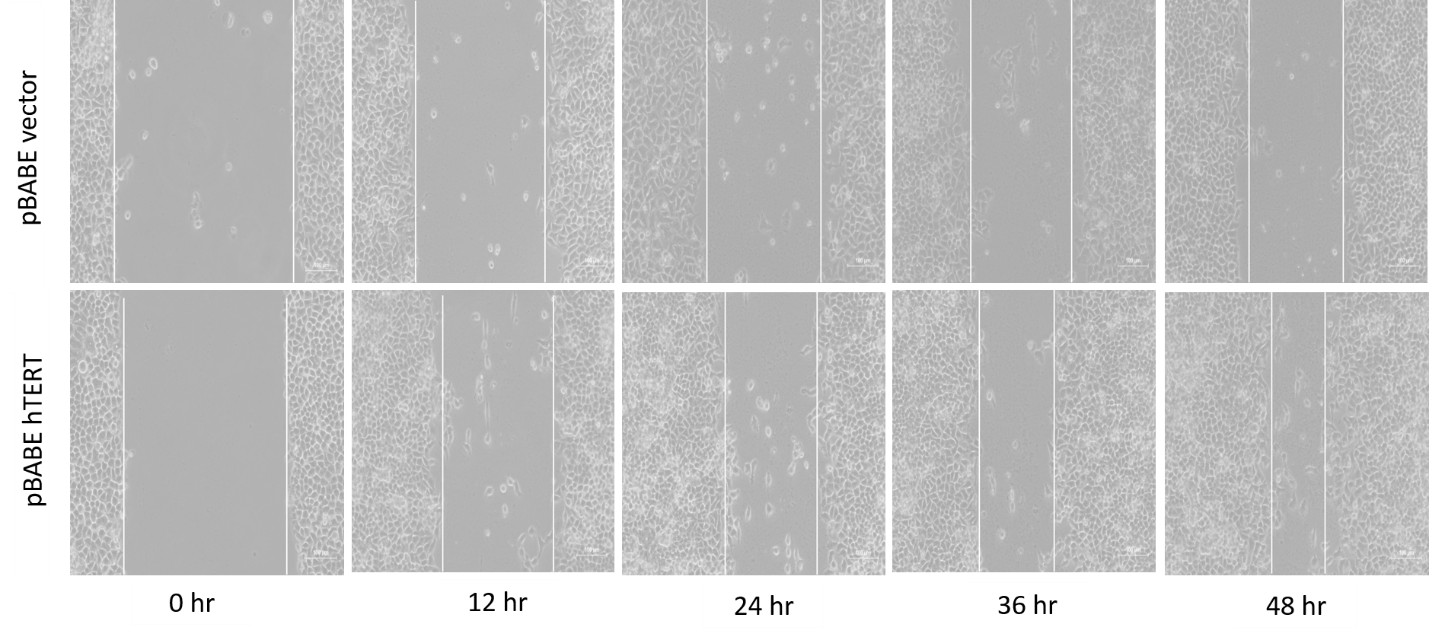


Fig D: Wound healing assay was performed in hTERT overexpressing U2OS cells.

Table F: Distance migrated (µm) value which is used to make graph in hTERT overexpressing HeLa cells. Wound healing assay was performed in hTERT overexpressing U2OS cells. Calculated distance are given in the table.

| Time | pBABE Vector | pBABE hTERT |
| --- | --- | --- |
| 0 hr | 563 | 520 |
| 12hr | 428 | 367 |
| 24 hr | 409 | 324 |
| 36 hr | 388 | 244 |
| 48 hr | 358 | 175 |

Fig E: Two dimensional gel electrophoresis of vector expressed U2OS cells-


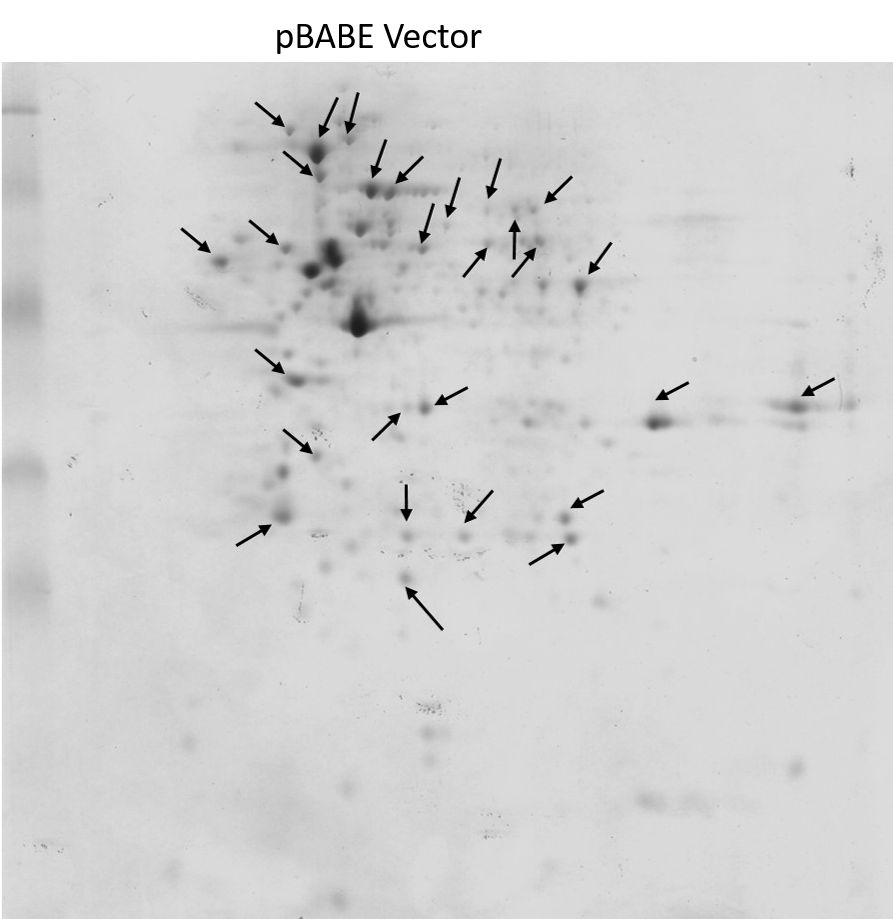


Fig F: Two dimensional gel electrophoresis of hTERT overexpressing U2OS cells.


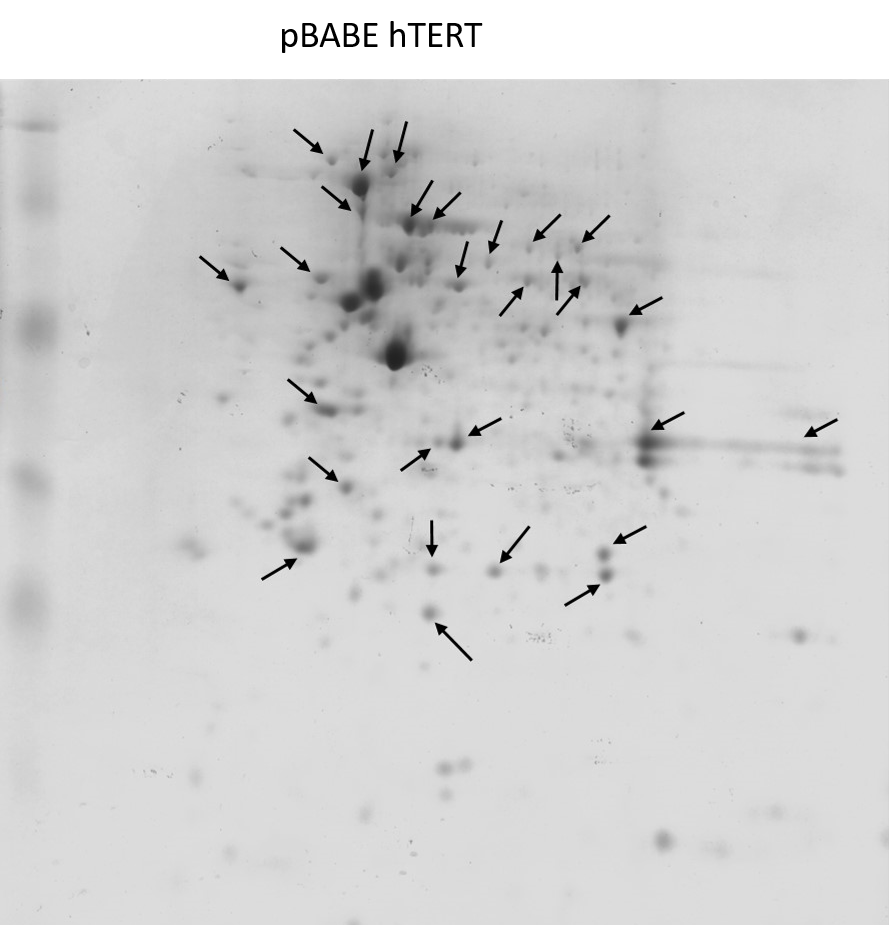


Fig E & F: Two dimensional gel electrophoresis was performed in Vector and hTERT overexpressing U2OS cells

Fig G: Two dimensional gel electrophoresis of vector expressed HeLa cells.


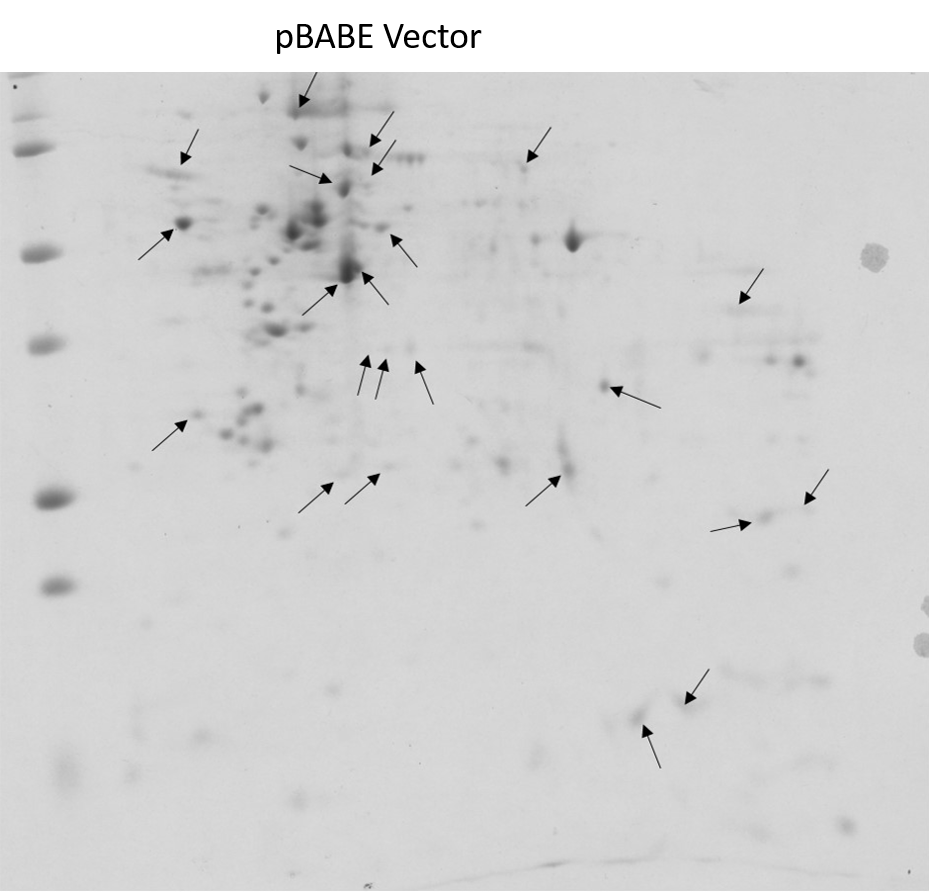


Fig H: Two dimensional gel electrophoresis of hTERT overexpressing HeLa cells.


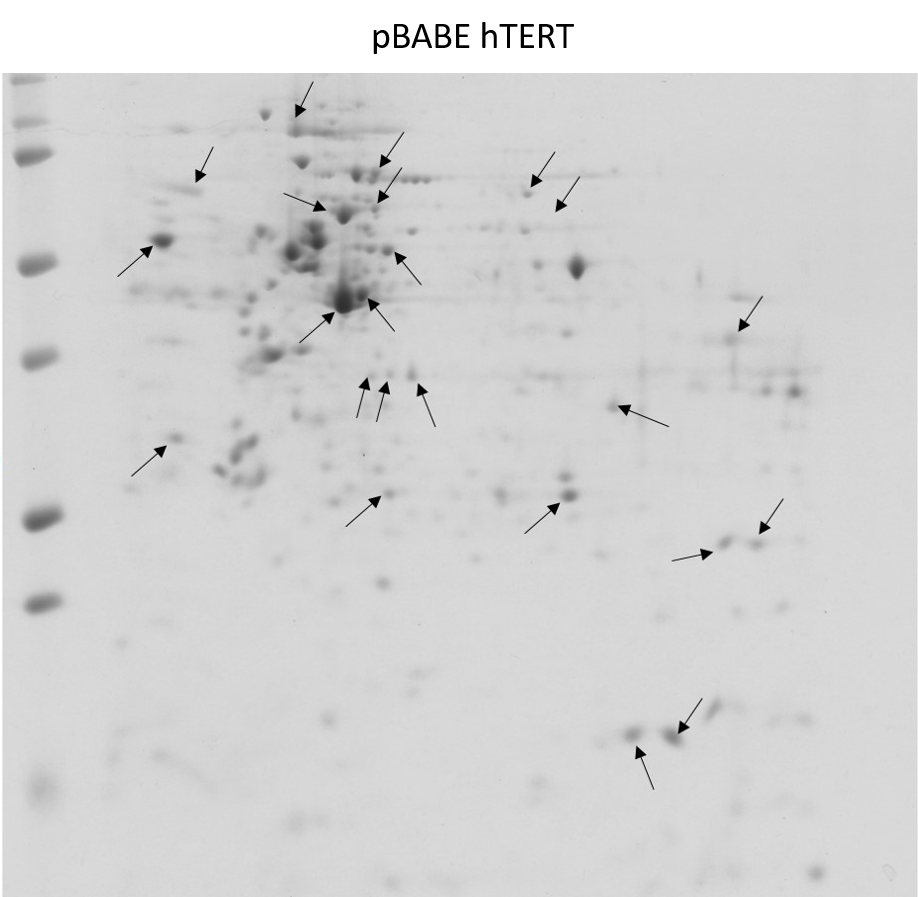


Fig G & H: Two dimensional gel electrophoresis was performed in vector and hTERT overexpressing HeLa cells.

Table G: Real-time data of Hsp60 in U2OS cells. Real-time PCR was performed to check the upregulation of Hsp60 in U2OS cells. Fold change values are given in the table.

| Name | Fold Change Value | ΔCт SE |
| --- | --- | --- |
| pBABE Vector U2OS | 1 | 0.064321 |
| pBABE hTERT U2OS | 1.563 | 0.044345 |

Table H: Real time data of Hsp70 in U2OS cells. Real-time PCR was performed to check the upregulation of Hsp70 in U2OS cells. Fold change values are given in the table.

| Name | Fold Change Value | ΔCт SE |
| --- | --- | --- |
| pBABE Vector U2OS | 1 | 0.068541 |
| pBABE hTERT U2OS | 3.987971 | 0.050416 |

Table I: Real-time data of Hsp60 in HeLa cells. Real-time PCR was performed to check the upregulation of Hsp70 in HeLa cells. Fold change values are given in the table.

| Name | Fold Change Value | ΔCт SE |
| --- | --- | --- |
| pBABE Vector U2OS | 1 | 1.259533 |
| pBABE hTERT U2OS | 113.176 | 0.203185 |

Table J: Real time data of Hsp70 in HeLa cells. Real-time PCR was performed to check the upregulation of Hsp60 in HeLa cells. Fold change values are given in the table.

| Name | Fold Change Value | ΔCт SE |
| --- | --- | --- |
| pBABE Vector U2OS | 1 | 0.180376 |
| pBABE hTERT U2OS | 1.817661 | 0.045062 |

Fig I: Western blotting of Hsp60 and Hsp70 in U2OS and HeLa cells following hTERT overexpression-


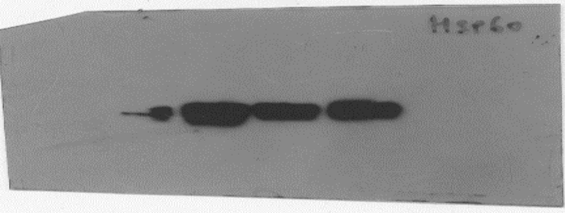


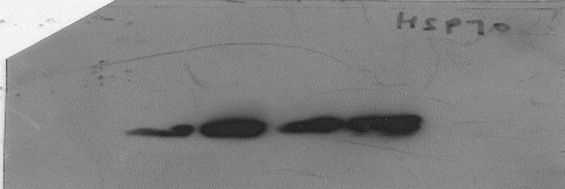


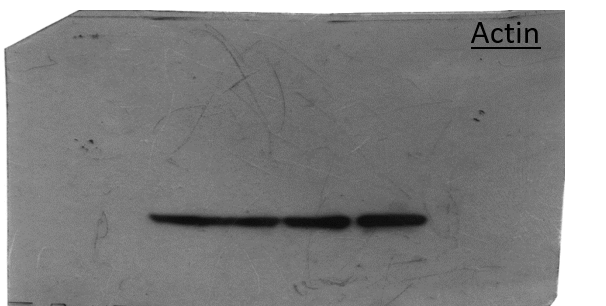


Fig I: Western blotting is performed to confirm upregulation of Hsp60 and Hsp70 in Hela and U2OS cells.

Table K: Distance migrated (µm) value which is used to make graph of Hsp60 in HeLa cells. : ImageJ software was used to calculate change in the expression of protein. Fold change values are given in the table.

| Name | Fold Change Value | Standard error of the mean |
| --- | --- | --- |
| pBABE Vector HeLa | 1 | 0.10645 |
| pBABE hTERT HeLa | 9.803285 | 0.13956 |

Table L: Distance migrated (µm) value which is used to make graph of Hsp60 in U2OS cells. ImageJ software was used to calculate change in the expression of protein. Fold change values are given in the table.

| Name | Fold Change Value | Standard error of the mean |
| --- | --- | --- |
| pBABE Vector HeLa | 1 | 0.06450 |
| pBABE hTERT HeLa | 1.247048 | 0.04956 |

Table M: Distance migrated (µm) value which is used to make graph of Hsp70 in HeLa cells. : ImageJ software was used to calculate change in the expression of protein. Fold change values are given in the table.

| Name | Fold Change Value | Standard error of the mean |
| --- | --- | --- |
| pBABE Vector HeLa | 1 | 0.07310 |
| pBABE hTERT HeLa | 1.992216 | 0.16956 |

Table N: Distance migrated (µm) value which is used to make graph of Hsp70 in U2OS cells. : ImageJ software was used to calculate change in the expression of protein. Fold change values are given in the table.

| Name | Fold Change Value | Standard error of the mean |
| --- | --- | --- |
| pBABE Vector HeLa | 1 | 0.04171 |
| pBABE hTERT HeLa | 1.447306 | 0.07337 |

Table O: Real time data of Hsp90 in U2OS cells. Real-time PCR was performed to check the upregulation of Hsp90 in U2OS cells. Fold change values are given in the table.

| Name | Fold Change Value | ΔCт SE |
| --- | --- | --- |
| pBABE Vector U2OS | 1 | 0.077027 |
| pBABE hTERT U2OS | 1.840021 | 0.066457 |

Fig J: Western blotting of Hsp90 in U2OS cells following hTERT overexpression-


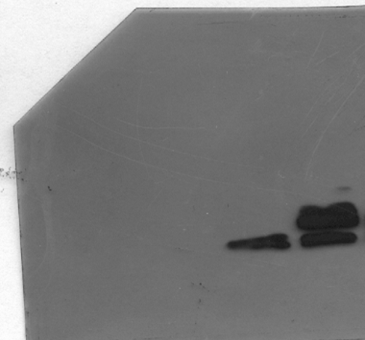

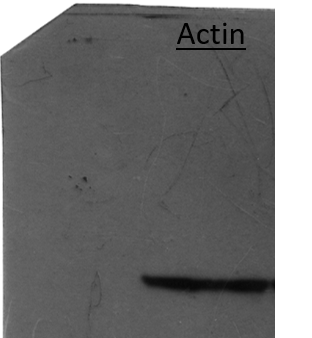


Fig J: Western blotting is performed to confirm upregulation of Hsp90 in U2OS cells.

Table P: Real time data of GAPDH in U2OS cells. Real-time PCR was performed to check the upregulation of GAPDH in U2OS cells. Fold change values are given in the table.

| Name | Fold Change Value | ΔCт SE |
| --- | --- | --- |
| pBABE Vector U2OS | 1 | 0.091948 |
| pBABE hTERT U2OS | 2.857953 | 0.072147 |

Table Q: Real time data of GAPDH in HeLa cells. Real-time PCR was performed to check the upregulation of GAPDH in HeLa cells. Fold change values are given in the table.

| Name | Fold Change Value | ΔCт SE |
| --- | --- | --- |
| pBABE Vector U2OS | 1 | 0.097107 |
| pBABE hTERT U2OS | 1.33832 | 0.029174 |

Fig K: Western blotting of GAPDH in U2OS and HeLa cells following hTERT overexpression-


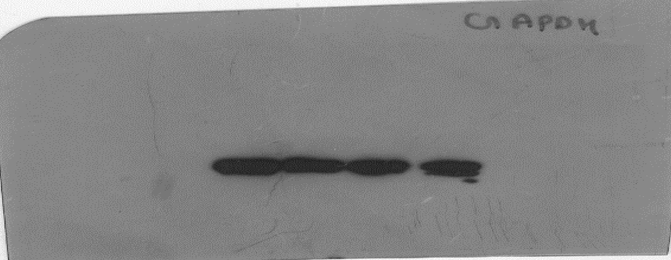


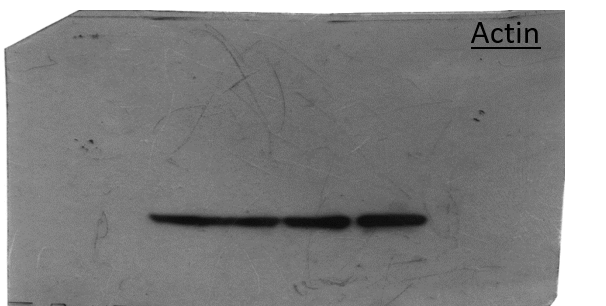


Fig K: Western blotting is performed to confirm upregulation of Hsp90 in U2OS cells.

Table R: Densitometric quantification of GAPDH in U2OS cells. ImageJ software was used to calculate change in the expression of protein. Fold change values are given in the table

| Name | Fold Change Value | Standard error of the mean |
| --- | --- | --- |
| pBABE Vector HeLa | 1 | 0.06987 |
| pBABE hTERT HeLa | 1.013572 | 0.07635 |

Table S: Densitometric quantification of GAPDH in HeLa cells. ImageJ software was used to calculate change in the expression of protein. Fold change values are given in the table

| Name | Fold Change Value | Standard error of the mean |
| --- | --- | --- |
| pBABE Vector HeLa | 1 | 0.05976 |
| pBABE hTERT HeLa | 1.028222 | 0.03997 |
